# Effect of substrate (ZnO) morphology on enzyme immobilization and its catalytic activity

**DOI:** 10.1186/1556-276X-6-450

**Published:** 2011-07-13

**Authors:** Yan Zhang, Haixia Wu, Xuelei Huang, Jingyan Zhang, Shouwu Guo

**Affiliations:** 1 National Key Laboratory of Micro/Nano Fabrication Technology, Key Laboratory for Thin Film and Microfabrication of the Ministry of Education, Research Institute of Micro/Nano Science and Technology, Shanghai Jiao Tong University, Shanghai 200240, PR China; 2 State Key Laboratory of Bioreactor Engineering, School of Pharmacy, East China University of Science and Technology, Shanghai, 200237, PR China

## Abstract

In this study, zinc oxide (ZnO) nanocrystals with different morphologies were synthesized and used as substrates for enzyme immobilization. The effects of morphology of ZnO nanocrystals on enzyme immobilization and their catalytic activities were investigated. The ZnO nanocrystals were prepared through a hydrothermal procedure using tetramethylammonium hydroxide as a mineralizing agent. The control on the morphology of ZnO nanocrystals was achieved by varying the ratio of CH_3_OH to H_2_O, which were used as solvents in the hydrothermal reaction system. The surface of as-prepared ZnO nanoparticles was functionalized with amino groups using 3-aminopropyltriethoxysilane and tetraethyl orthosilicate, and the amino groups on the surface were identified and calculated by FT-IR and the Kaiser assay. Horseradish peroxidase was immobilized on as-modified ZnO nanostructures with glutaraldehyde as a crosslinker. The results showed that three-dimensional nanomultipod is more appropriate for the immobilization of enzyme used further in catalytic reaction.

## Introduction

The composition, size, and surface characteristics of substrates have been considered as the important factors that affect the physical/chemical properties of the immobilized enzymes [1-3]. However, for conventional bulk solid substrates, such as glass slides [4], polymer monoliths [5,6], and silica beads [3], the control on their morphologies and functionalizing their surfaces are usually laborious. Nanoscaled materials, such as metal [7] and metal oxide nanospheres [8], carbon nanotubes [9,10], graphene oxide nanosheets [11], have also been utilized as the substrates for enzyme immobilization. It has been demonstrated that the size of nanostructured materials might play significant roles to regulate the catalytic activity of the immobilized enzymes [3]. The preparation of nanostructured materials with controlled chemical composition, size, morphology, and the surface functionalization have witnessed great progressive achievements during the last two decades [12,13]. However, the systematic study of the effects of the morphology of the nanoscale substrates on the enzyme immobilization remains to be expanded. ZnO nanocrystals have unique physical/chemical properties and pronounced biocompatibility, which are beneficial for many practical applications. In addition, a variety of nanostructures of ZnO, such as nanospheres [14], nanowires [15], nanorods [16], nanonails [17], nanotubes [18], nanotetrapods [19], nanotablets [20], and nanoflowers [21], have been prepared successfully. Therefore, ZnO nanocrystals are ideal materials to study the effect of the morphology of the substrate on the catalytic efficiency of the immobilized enzymes. Horseradish peroxidase (HRP) was used as a model enzyme because it has been wildly studied and used in many fields, such as organic syntheses [22], phenol removal [23], biosensor, and drug delivery [24].

In this study, we report the effects of the morphology of the ZnO nanocrystals on the enzyme immobilization. ZnO nanocrystals with different morphologies, including nanosphere, nanodisk, and nanomultipod, were fabricated simply through a hydrothermal procedure. The surface of ZnO nanocrystal was functionalized with the amino groups using 3-aminopropyltriethoxysilane (APTES) and tetraethyl orthosilicate (TEOS) [25]. Glutaraldehyde was used as a crosslinker to immobilize the HRP enzyme molecules on the surface of as-modified ZnO nanocrystals. Then the enzyme loading and catalytic activity were evaluated.

## Results and discussion

### ZnO nanocrystals with different morphology

**Figure 1:**
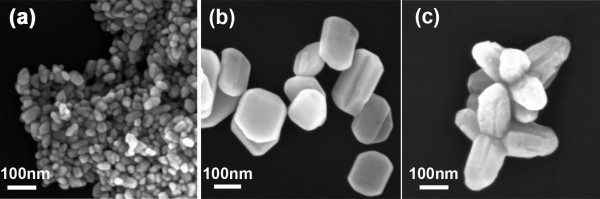
**SEM images of ZnO nanocryatals**. **(a)** Nanospheres, **(b)** nanodisks, and **(c)** nanomultipods prepared using mixtures of methanol and water with different volume ratios of 10:0, 1:1 and 1:9 as solvents, respectively.

**Figure 2:**
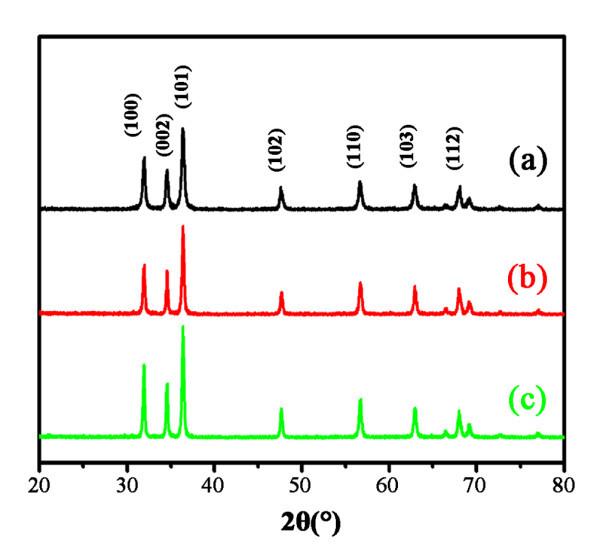
**XRD patterns of ZnO nanocryatals**. **(a)** Nanospheres, **(b)** nanodisks, and **(c)** nanomultipods.

Figure 1 shows the SEM images of ZnO nanocrystals with different morphologies. The nanospheres, with diameter of approximately 30 nm, were obtained when pure methanol was used as the hydrothermal reaction solvent as shown in Figure 1a. When the ratio of methanol to water was adjusted to 1:1, ZnO nanodiscs, approximately 85 nm in width and 25 nm in thickness, were acquired as shown in Figure 1b. Figure 1c shows the ZnO nanomultipods composed of several rods of 100 nm in diameter and 200 nm in length when the methanol/water ratio reaches 1:9. This illustrates that the ratio of methanol/water is certainly a crucial factor to control on the morphology of ZnO nanocrystals. Although the morphologies of aforementioned ZnO nanocrystals are different, the XRD patterns are all well consistent with the standard wurtzite ZnO structure as shown in Figure 2. Thus, the effect of crystal form on the surface modification and enzyme immobilization can be neglected.

### Surface modification of ZnO nanocrystals

**Figure 3:**
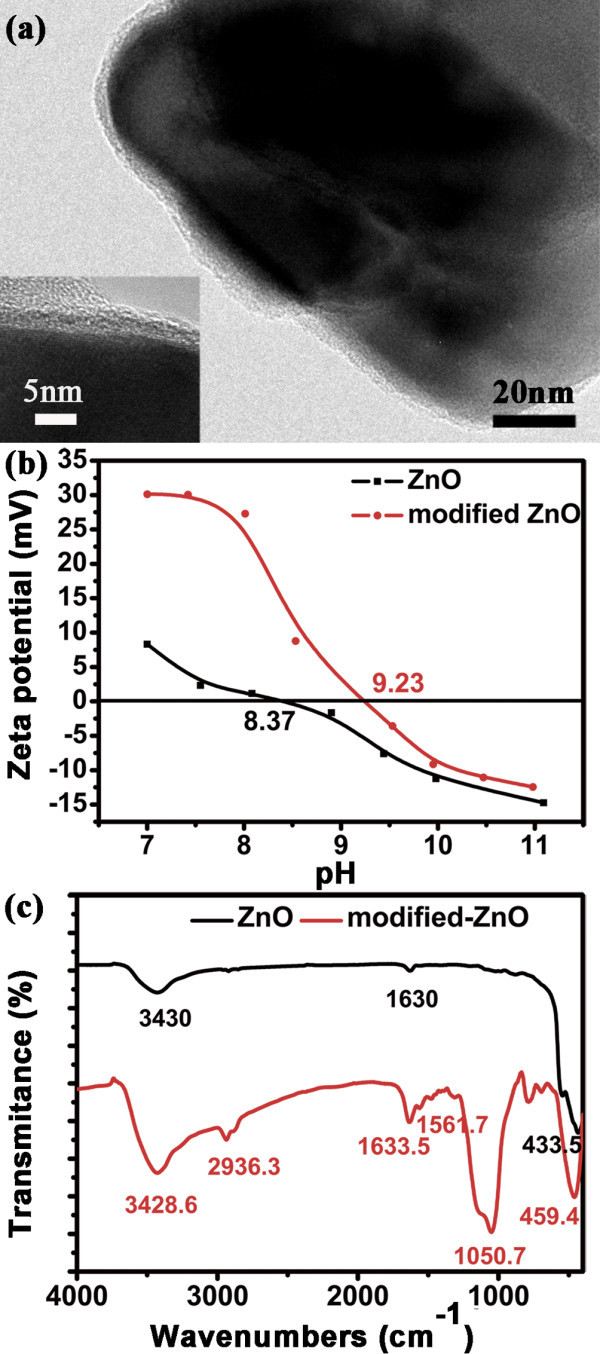
**The surface functionality of ZnO nanodisks before and after modification**. **(a) **The TEM images of ZnO modified by TEOS:APTES (1:4 in volume). **(b) **Zeta-potential curves, and **(c) **FT-IR spectra of ZnO nanodisks before and after modification.

In order to immobilize enzyme, the surface of ZnO nanocrystals were functionalized with amino groups using APTES/TEOS. Figure 3a shows a typical image of coated ZnO nanodiscs, where a thin film with uniform thickness of 2 nm formed on the surface. Comparing the Zeta-potentials of the bare ZnO and the as-modified ZnO nanocrystals (Figure 3b), it can be deduced that the surface electrostatic state of ZnO nanocrystals was changed. The surface groups of the modified ZnO were further characterized by FT-IR spectra. Through comparing the FT-IR spectra before and after modification in Figure 3c, except for a few peaks at 3430, 1630, and 433 cm^-1 ^corresponding to water (moisture) and ZnO nanocrystal, the peaks at 2936.3 and 2872 cm^-1 ^of the C-H stretching vibration [26], and the peaks at 1330 and 1560 cm^-1 ^of the stretching vibration of C-N and bending vibration of N-H can be found. In addition, the strong absorption peaks at 3428.6 and 1633.5 cm^-1^, assigned to N-H bending vibrations, are overlapped with the bending vibration of the absorbed H_2_O [27]. These results confirmed the presence of amino groups on the ZnO nanocrystals surface.

**Figure 4:**
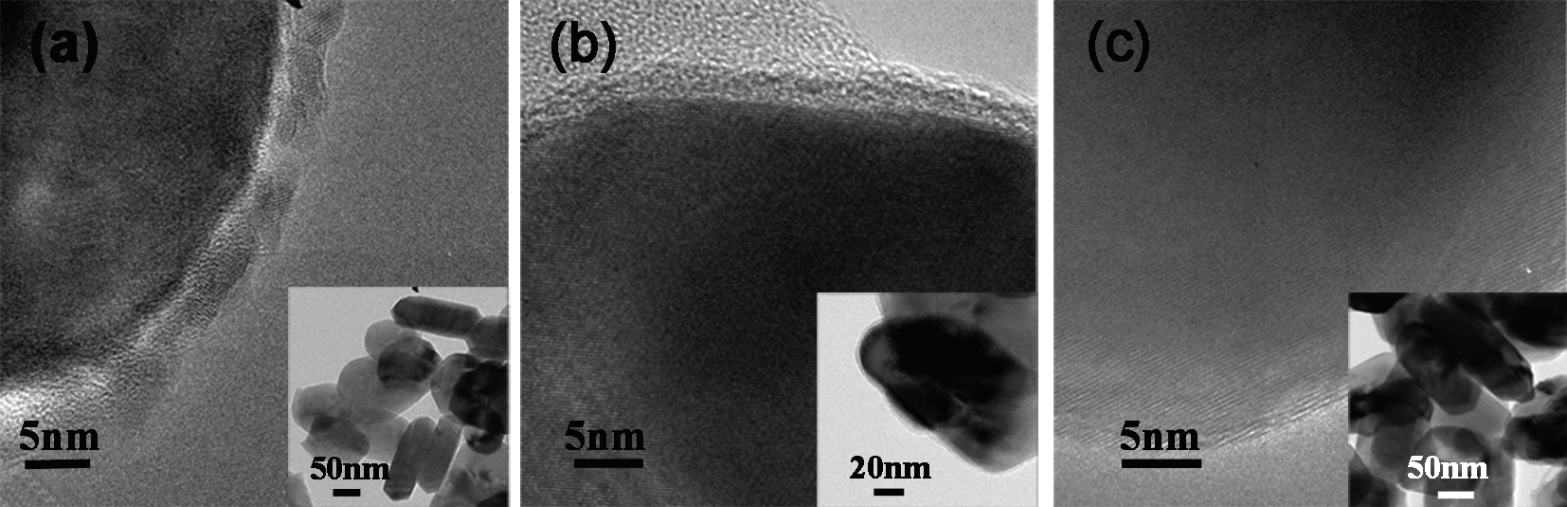
**TEM images of amino group modified ZnO nanodisks**. **(a-c) **TEM images of modified ZnO nanodisks with different TEOS:APTES ratios of 1:1, 1:4, and 1:10.

The amount of amino groups and the thickness of the coating layer can be controlled by adjusting the ratio of TEOS to APTES. When the ratio of TEOS to APTES was 1:1, a coating layer of approximately 2 nm can be generated on the surface of ZnO nanocrystal, but, at the same time, lots of isolated SiO_2 _nanocrystals were formed, as shown in Figure 4a. When the ratio of TEOS to APTES was 1:4, a layer with uniform thickness of about 2 nm was formed as shown in Figure 4b. When the ratio of TEOS to APTES was decreased to 1:10, no fully covered coating layer can be generated, as shown in Figure 4c. According to the standard curve of glycine obtained by Kaiser Assay, the amount of amino groups on the surface of ZnO nanocrystals was deduced. When the ratios of TEOS to APTES were 1:1, 1:4, and 1:10 used for the surface modification, the amounts of amino groups on the surface of ZnO nanodisks were 0.03, 0.07, and 0.02 mmol/g, respectively. These results show that the ratio of TEOS to APTES used for the surface modification determines the uniformity of the coating layer as well as the amount of amino groups.

**Figure 5:**
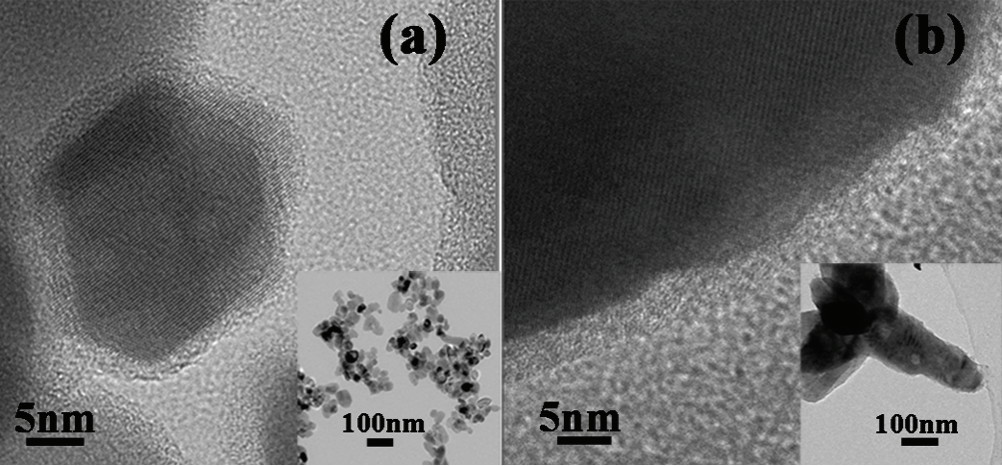
**TEM images of amino group-functionalized ZnO nanocrystals**. **(a) **Nanospheres and **(b) **nanomultipods using TEOS and APTES with the ratio of 1:4 in volume.

The aforementioned procedure was also performed on the surface modifications of ZnO nanospheres and nanomultipods with TEOS to APTES ratio of 1:4. Figure 5 depicts the TEM images of the nanosphere and nanomultipod after the modification. Similar to the nanodisks, there are thin coating layers formed both on nanospheres and nanomultipods. The amounts of amino groups on the surfaces of ZnO nanospheres and nanomultipods modified with TEOS to APTES ratio of 1:4 were also measured, which are 0.127 and 0.044 mmol/g, respectively. The specific surface areas of ZnO nanospheres, nanodisks, and nanomultipods are 33.59, 11.99, and 11.85 m^2^/g, respectively, which were measured using Brunauer-Emmett-Teller (BET) method. Thus, considering the surface areas of different morphologies, the surface densities of amino groups on ZnO nanospheres, nanodisks, and nanomultipods were calculated, which are determined to be 3.78, 5.94, and 3.71 μmol/m^2^, respectively.

### The effects of morphologies of ZnO nanocrystals on HRP immobilization and their activity

**Figure 6:**
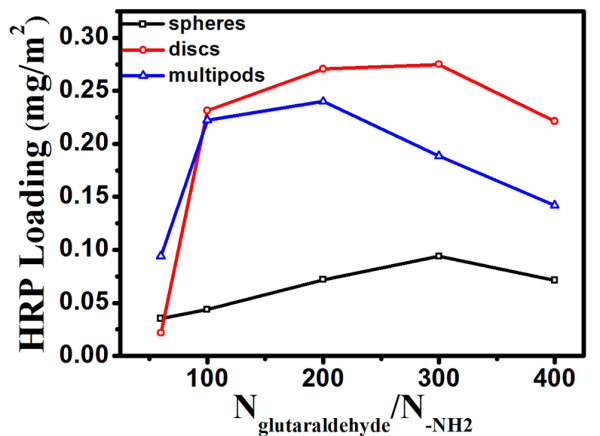
**The enzyme loadings on different morphologies of ZnO nanocrystals**. The loadings of HRP with different ratios of glutaraldehyde and amine groups on the surface of the modified ZnO nanocrystals.

The two aldehyde groups (-COH) of glutaraldehyde can bond separately to the amino groups of HRP and as-modified ZnO [28], and thus, glutaraldehyde was used as a crosslinker to immobilize HRP molecules on the modified ZnO nanocrystal surfaces. As shown in Figure 6, the highest loadings of HRP on the ZnO nanospheres, nanodisks and nanomultipods were 0.094, 0.275, 0.240 mg/m^2^, respectively From Figure 6, we find that the immobilization of HRP on ZnO nanomultipods can reach the highest loading at the lowest ratio of glutaraldehyde to amino groups. The maximum loading of HRP on ZnO nanomultipods was higher than that on the nanospheres, but, as high as that on the nanodisks, even if the surface density of amino groups on ZnO nanomultipods was relatively lower than that of the other two.

The catalytic activity of the HRP immobilized on different ZnO nanocrystals was assayed through phenol oxidation reaction. Soluble HRP was also characterized as a control. Their kinetic parameters were obtained from the Lineweaver-Burk equation, and the data are summarized in Table 1. 1/*K_m_*, which express the affinity of phenol compounds to HRP, and the *K_m _*of the HRP immobilized on ZnO nanospheres, nanodisks and nanomultipods were tagged as *K_m_*(*s*), *K_m_(d)*, and *K_m_*(*m*), respectively. Table 1 shows that *K_m_*(*s*) was unexpectedly higher than that of the free HRP, while *K_m_*(*d*) and *K_m_*(*m*) are close to that of free HRP suggesting that the substrate affinity to the HRP was not affected by the immobilization on these two ZnO materials. Thus, we can assume the morphology of the ZnO nanocrystals must be an important fact that affects the affinity of phenol compounds to HRP.

**Table 1:** The loadings and kinetic properties of the HRP immobilized on ZnO nanocrystals

sample	**Enzyme loading (mg/m**^**2**^**)**	*K_m _*(mM)	***K***_**cat**_**/*K_m _*(mM**^**-1 **^**s**^**-1**^)
Free HRP	-	6.24	80.13
HRP on ZnO nanospheres	0.094	23.06	0.78
HRP on ZnO nanodisks	0.275	3.95	1.09
HRP ZnO nanomultipods	0.240	2.42	1.28

The catalytic property of the immobilized HRP was further characterized by the catalytic efficiency (*K*_cat_/*K_m_*). The catalytic efficiency of immobilized HRP was much lower than that of free HRP, which coincide with the report of the literature [29]. As shown in Table 1, the catalytic efficiency values of immobilized HRP on the nanospheres, nanodisks and nanomultipods were 0.78, 1.09, 1.28 mM^-1 ^s^-1^, respectively. Through comparing the *K*_cat_/*K_m _*value of the HRP immobilized on three ZnO nanocrystals with different morphologies, the nanomultipod ZnO nanocrystals are apparently favorable for enzyme immobilization.

Those all may due to the three dimensional structural feature of nanomultipods, which could affect the enzyme interaction with the immobilized substrate and conformation of the enzyme, and result in increasing the enzyme loading on the solid substrate and catalytic efficiency. Because the nanomultipods have several pods, and the spaces among the pods are limited, the glutaraldehyde is difficult to self-polymerize, and the amino groups on the multipods are more efficiency to immobilize HRP. While, nanospheres and nanodisks have opened spaces, glutaraldehyde tends to polymerize. Thus, HRP loadings on nanospheres and nanodisks need more glutaraldehyde to reach the maximum loadings. Due to the opened structure and lower surface density of amino groups, the loading of HRP on nanospheres is lowest. Thus, HRP loadings on nanospheres and nanodisks need more glutaraldehyde to reach the maximum loadings. Compared with nanodisks and nanospheres, the HRP loading on ZnO nanomultipods reached the highest when its glutaraldehyde to amino groups ratio was lower, which indicates that every HRP molecule needs less glutaraldehyde to immobilize HRP on the surface of ZnO nanocrystal and less conformation happened. Therefore, the 3D structure of nanomultipods and the lowest ratio of *N*_glutaraldehyde_/*N*-_NH2 _may result in the stabilization of HRP immobilized on nanomultipods, which indicates that the 3D nanomultipod is more appropriate for the immobilization of enzyme and its catalytic efficiency.

## Conclusions

ZnO nanocrystals with different morphologies, including nanosphere, nanodisk, and nanomultipod, were prepared via hydrothermal reactions using the mixtures of methanol/water with different volume ratios. The surfaces of ZnO nanocrystals were modified with amino groups using TEOS and APTES to study the effect of the morphology of the materials to the enzyme immobilization. The surface density of the amino groups and the thickness of coating were controlled by tuning the ratio of TEOS to APTES. It was demonstrated when the ratio of TEOS to APTES was 1:4, a layer of uniform thickness of approximately 2 nm can be generated on surface of the ZnO nanocrystals.

HRP molecules were immobilized on the modified ZnO nanocrystal surfaces using glutaraldehyde as a crosslinker. It was illustrated that the enzyme loading on the ZnO nanostructure was in the order of nanospheres < nanomultipod < nanodisks, while the nanomultipod reached the highest loading when its glutaraldehyde to amino groups ratio was lower than the other two, causing less conformation change of HRP on the ZnO surface, leading to a higher catalytic efficiency. In brief, the 3D nanomultipod is more appropriate for the immobilization of enzyme and for being used in catalytic reaction than the other two, which has great implications for the many ongoing studies of enzyme immobilization and applications of the immobilized enzymes.

## Methods

### Materials

Zn(Ac)_2_·2H_2_O, (CH_3_)_4_NOH (25%), Glutaraldehyde (25%), phenol (99%), 4-aminoantipyrine (4-AAP), methanol, and H_2_O_2 _(30%) were purchased from Sinopharm Chemical Reagent Company, Shanghai, China. APTES (≥98.0%) was bought from Sigma-Aldrich, USA. TEOS was obtained from Linfeng Chemical Reagent Company, Shanghai, China. HRP was purchased from Majorbio Biotech Company, USA. All reagents were used as-received.

### Synthesis of ZnO nanocrystals

The ZnO nanocrystals were prepared through a hydrothermal procedure using tetramethylammonium hydroxide as a mineralizing agent. The control on the morphology of ZnO nanocrystals was achieved by varying the ratio of CH_3_OH to H_2_O, which were used as solvents in the hydrothermal reaction system. In a typical experiment, Zn(Ac)_2_·2H_2_O (5 mmol) was dissolved in 15 mL of the mixture of methanol and water with different volume ratios in a flask under vigorous stirring. Then, 15 mL of (CH_3_)_4_NOH was dropped into the flask as mineralizing agent. The as-obtained turbid suspension was transferred into a Teflon inner reactor of stainless-steel autoclave. The autoclave was heated at 200°C for 24 h for the hydrothermal reaction. The reaction solution was then cooled down to room temperature and the solid product was separated from the reaction mixture by centrifugation (4000 rpm), and was washed with ethanol and water alternately, each for three times.

### Characterization of the ZnO nanocrystals

The size, morphology, BET surface area, and crystallinity of the ZnO nanocrystals before and after the surface modification were characterized using scanning electron microscope (Zeiss ultra 55, Germany), transmission electron microscopy (TEM) (JEM-2100, Japan), accelerated surface area, porosimetry system (Micromeritics ASAP 2010 M+C, USA), and X-ray powder diffraction (XRD) (BRUKER-AXS, Germany). The surface functionalities of the ZnO nanocrystals after the modification were studied using FT-IR with a Nicolet 5700 Fourier transform infrared spectrometer (Thermo Electron, USA), and Zeta potentials obtained on Nicomp 380/ZLS (America).

### Surface functionalization of ZnO nanocrystals

A typical surface functionalization process was as follows.

In general, 100 mg of ZnO nanocrystals was suspended into 20 mL of ethanol (pH = 10.8) under sonication. Then, 15 μL of TEOS and 60 μL of APTES were added into the ethanol solution, and the mixture was stirred for 5 h. The solid product was filtered and washed with ethanol, and then dried at room temperature. The amounts of amino groups on the surface of ZnO nanocrystals were measured by Kaiser Assay. At first, the standard curve of glycine obtained by Kaiser Assay. Glycine were mixed with 2 mL of acetate buffer (0.6 M, pH = 4.5) and 2 mL of 10 mg/mL ninhydrin ethanol solution. The mixture was treated at 90°C for 20 min. After centrifugation at 10,000 rpm for 3 min, the amino groups' concentration in the supernatant was monitored spectrophotometrically 565 nm, and then, 10 mg of modified ZnO was utilized to monitor spectrophotometrically as glycine. Then, the amount of amino groups was calculated based on the standard curve of glycine.

100 mg modified ZnO was suspended into 20 mL pH 7.0 0.1 M phosphate buffers, and then 750 μL of 25% glutaraldehyde solution was added to the mixture. The mixture was incubated at room temperature, 200 rpm for 2 h. After filtration and washing with the phosphate buffer, the samples were utilized to immobilize HRP.

### HRP immobilization

For HRP immobilization, 100 mg of functionalized ZnO nanocrystals was added into 1 mL, 0.1 M, and pH 7.0 of potassium phosphate buffer containing 100 μg of HRP. The mixture was incubated at 4°C for 2 h with 240 rpm shaking, and then centrifuged at 10,000 rpm for 3 min. The supernatant was collected. The sediments were centrifuged and rinsed alternately three times with 0.1 M, pH 7.0 phosphate buffer solution to remove non-specifically adsorbed enzymes. The solid was stored at 4°C for further measurements. The supernatants were employed to determine the enzyme loading on modified ZnO.

### Characterization of the immobilized HRP

The enzyme loading is obtained by subtracting the amount of the left HRP in the supernatant from the total HRP added. Free HRP and the immobilized HRP activity was assayed by colorimetric method using 4-AAP as previously described [2]. In brief, the immobilized enzyme was added into 1 mL of 0.1 M, pH 7.0, phosphate buffer which contained 60 mM of phenol, 14.38 mM of 4-AAP, and 1.21 mM of hydrogen peroxide, and then reacted at 30°C for 3 min. The initial catalytic reaction rates of the enzyme in the supernatant and the immobilized enzyme were determined by measuring the UV absorbance of the reaction mixture at 510 nm. The double reciprocal plots of the rates and substrate concentrations were plotted to obtain *K_m _*and *K*_cat _according to the Lineweaver-Burk equation.

## Abbreviations

4-AAP: 4-aminoantipyrine; APTES: aminopropyltriethoxysilane; BET: Brunauer-Emmett-Teller; HRP: horseradish peroxidase; TEM: transmission electron microscopy; TEOS: tetraethyl orthosilicate; XRD: X-ray powder diffraction; ZnO: zinc oxide.

## Competing interests

The authors declare that they have no competing interests.

## Authors' contributions

YZ conducted the experiments and performed data analysis. XH helped in designing the immobilized enzyme test. JZ participated in the design and helped in compiling the data of immobilization enzyme interpretation. HW helped during the most of operation and data interpretation of analytic equipments used. SG conceived basic idea of this technique and supported the organization of this article.
